# Effect of acupuncture on patients with insomnia: study protocol for a randomized controlled trial

**DOI:** 10.1186/1745-6215-15-403

**Published:** 2014-10-23

**Authors:** Kyung-Hun Han, Sang-Young Kim, Sun-Yong Chung

**Affiliations:** Behavioral Research Center, Korea University, 1, 5-Ka Anam-Dong, Sungbuk-ku, Seoul Korea; Department of Hwabyung/Stress Clinic, Kyung Hee University Korean Medicine Hospital at Gangdong, 149, Sangil-Dong, Gangdong-gu, Seoul Korea

**Keywords:** Insomnia, Acupuncture, Intradermal acupuncture, Clinical trial

## Abstract

**Background:**

Hypnotic drugs tend to be the dominant form of treatment of insomnia, but these come with a number of reported side effects. Acupuncture has been studied as an alternative, resulting in a rising need for methodological research towards verifying its efficacy as insomnia treatment.

**Methods/Design:**

We describe a proposal for a single-center, patient-assessor-blinded, randomized controlled trial with two parallel arms. A total of 38 patients complete screening tests at the first visit, are registered into the clinical trial, and then randomly assigned to the experimental or sham control groups (19 patients for each group). All subjects are clinical insomnia patients who score a 6 or above on the Pittsburgh Sleep Quality Index (PSQI) and meet all inclusion criteria. All subjects are treated with acupuncture and intradermal acupuncture (IDA) three times during the first week. Five sham acupoints are used in the control group. In the experimental group, five real acupoints (PC6, SP6, HT7, KI6, and BL62) are used unilaterally in turn. Sham acupoints are over 1 cm away from each real acupoint.

The primary outcomes are the scores on the Insomnia Severity Index (ISI) and PSQI. Secondary outcomes are the sleep log, the Beck Depression Inventory (BDI), the State-Trait Anxiety Inventory (STAI), the World Health Organization Quality of Life Abbreviated Version (WHOQOL-BREF), the Korean-Auditory Verbal Learning Test (K-AVLT), the Digit Span Test (DS), Event Related Potentials (ERPs) and heart rate variability (HRV) to assess emotional states, sleep quality, cognitive functioning, and electro-physiological changes.

Subjects are assessed at three time points: baseline, post-treatment and follow-up. The duration of the clinical trial is 18 days.

**Discussion:**

To study the enhancement of the effectiveness of acupuncture for insomnia, we test the intradermal acupuncture method, which is performed continuously on the subject’s skin and stimulated at home by the subject every night. In the trial, objective measurements including ERPs and HRV are used to evaluate states of cognition and autonomic nervous system functioning and subjective self-report questionnaires assess insomnia symptoms.

'Sham’ acupuncture points provided by STRICTA are used for the control group.

**Trial registration:**

ClinicalTrials.gov: NCT01956760, registered 5 September 2013.

## Background

Insomnia is commonly defined as a state of being disturbed during daytime activities due to poor sleep quality caused by difficulties in sleep initiation or maintenance despite being in a proper sleep-inducing environment. According to the *International Classification of Sleep Disorders*, *Second Edition* (ISDC-2), insomnia can be categorized as acute insomnia, inadequate sleep hygiene, psychophysiological insomnia (primary insomnia), idiopathic insomnia, paradoxical insomnia, and insomnia associated with a medical condition [1]. Approximately 35% of adults experience insomnia at least once in their lives and almost half suffer seriously. Several studies report that approximately 10% of adults are disrupted in their daily activities due to chronic insomnia [2, 3].

Benzodiazepines, nonbenzodiazepine sedatives, and melatonin agonists are the primary pharmacological treatments for insomnia. Secondary treatment includes antidepressants, diphenhydramine, antipsychotics, and barbiturates [4]. Although effective, these medications often cause side effects such as residual daytime sedation, drowsiness, dizziness, lightheadedness, cognitive impairment, motor incoordination, and dependence [5]. The current most prevalent non-pharmacological therapies for insomnia are sleep hygiene education [6] and cognitive and behavioral psychotherapy, but such treatments require long-term implementation [7].

The effect of acupuncture on insomnia is consistently emerging as an alternative treatment for insomnia. In a systematic review by Yeung *et al*., 20 randomized controlled trials were analyzed to assess the effect of acupuncture on insomnia patients by comparing them with control patients given a benzodiazepine, with results indicating that acupuncture was more effective. The study, however, has certain limitations including an undefined criterion for diagnosis, lack of randomization or blinded conditions, and small sample sizes [8]. Several other studies investigate the effect on acupuncture as a treatment for insomnia by comparing different acupuncture therapies, the effects of using and not using moxa, and comparison with placebo sleeping pills [9–11]. One of these studies shows that auricular acupuncture therapy is more effective for insomnia than two other therapies, and another suggests that acupuncture therapy with moxa is more effective than without [9, 10]. Cheuk *et al*., in a review of 33 clinical trials on the effects of acupuncture on insomnia, conclude that many trials indicate acupuncture treatment improves quality of sleep, but future trials need to be more methodologically rigorous, and that extant trials may be prone to the risk of bias coming from the definition of insomnia, participant characteristics, acupoints, and treatment regimen [12]. Despite the number of studies related to acupuncture and insomnia in the literature however, it is necessary for future trials to provide more methodologically sound evidence of acupuncture’s effect.

It is also is important to include an assessment of psychological/mood states, cognitive functions, and physiological states in future evaluations. According to the diagnostic criteria of insomnia defined by the *Diagnostic and Statistical Manual of Mental Disorders*, *Fourth Edition* (DSM-IV), insomnia is related to anxiety and depression, with many previous neuropsychological studies reporting that lack of sleep may lead to learning-memory disturbances, low attention with a descending level of concentration, and mood changes [13]. Regarding the pathophysiology of insomnia, several studies show that beta electroencephalogram (EEG) activities increase while alpha EEG activities decrease in patients with insomnia [14]. Other studies observed decreasing beta EEG activities in patients with insomnia after cognitive behavior therapy or treatment [15]. Some studies of Event Related Potentials (ERPs) report increasing N1 amplitude and decreasing N350 amplitude in insomnia patients compared to controls; these ERPs’ components are related to hyperarousal and inhibition or regulation of arousal [16, 17]. Additionally, a recent study shows that HF (high frequency) in heart rate variability (HRV) is more dominant than LF (low frequency) in patients with insomnia [18]. However, according to one review, there are no consistent results of HRV in studies of effect in acupuncture treatment, although HRV is a useful tool for observing how acupuncture treatment affects the autonomic nervous system related to sleep cycles [19].

Furthermore, relying on self-report measures - which are subjective and vulnerable to biases - to verify symptom reporting is inadequate. Changes in psychological, physiological, and cognitive functions should be observed using more objective methods, such as neuropsychological tests and electro-physiological measures.

## Methods/Design

### Hypothesis

Insomnia patients treated with acupuncture therapy would have their symptoms more effectively relieved than patients in a sham control group.

### Objectives

1. To examine significant improvement in the Insomnia Severity Index (ISI), the Pittsburgh Sleep Quality Index (PSQI), and sleep logs of insomnia patients [20, 21];
2. To compare changes in EEG and HRV brought on by acupuncture treatment;
3. To compare changes on the Beck Depression Inventory (BDI), the State-Trait Anxiety Inventory (STAI), the World Health Organization Quality of Life Abbreviated Version (WHOQOL-BREF), the Korean-Auditory Verbal Learning Test (K-AVLT), and the Digit Span Test (DS) from pre- to post-acupuncture treatments [22–26].

### Setting

The study will be performed at the Hwabyung/Stress Clinic in the Kyung Hee University East-West Neo Medical Center at Gangdong, Seoul, as a single-center, patient-assessor-blinded, parallel-group randomized controlled trial. Subjects will be recruited through advertisements on Internet websites, newspapers, and hospital bulletins. Subjects interested in participating in the trial shall be assessed according to inclusion and exclusion criteria by phone. Subjects who meet those criteria will be asked to visit the investigation site.

At the site first visit for screening meeting, subjects will be given more detailed information about the procedures of the study and asked to provide written informed consent if they wish to enroll. Candidate subjects will then complete the PSQI, with a score of 6 or above on the PSQI being used to diagnose subjects as clinical insomnia patients. At this point, inclusion and exclusion criteria will be reassessed, with patients satisfying those criteria being enrolled and registered into the clinical trial.

Registered patients shall be provided with a handout on sleep hygiene education that includes a brief account by the experimenter. They shall also be instructed to complete a sleep log for 2 days prior to their second visit to the center for the baseline evaluation and the first treatment, in order to see whether they had experienced insomnia recently and could properly keep their sleep logs. On the day of the second visit, an expert will check whether patients have completed their sleep logs correctly and re-conduct them on their sleep log writing methods if necessary. The patients will complete their sleep logs during 2 weeks from the second visit.

On the second visit day for the baseline evaluation and the first treatment, all patients will also complete the PSQI, ISI, BDI, STAI, K-AVLT, and the DS, with HRV measured at baseline (prior to first treatment), after the last treatment, and at the 1-week follow-up. EEG recordings will be used as objective measures to validate self-reported insomnia symptoms. ERPs will be measured at baseline and after the last treatment using an odd-ball task. Spontaneous EEGs will also be measured at baseline after the last treatment for spectral analysis. All subjects will complete the WHOQOL-BREF at baseline and at 1-week follow-up. They will also complete the Temperament Character Inventory (TCI) at baseline. All patients will also complete a sleep log every day from baseline to the 1-week follow-up [27]. The Credibility of Treatment Rating Scale (CTRS) will be administered to assess the maintenance of blind conditions after the second treatment [28].

Registered patients shall be randomly divided into the experimental and sham control groups, with an equal number of patients assigned to each group. Patients will then complete either the experimental or sham acupuncture treatment 3 times, with 2- to 3-day intervals, within 1 week.

**Figure 1:**
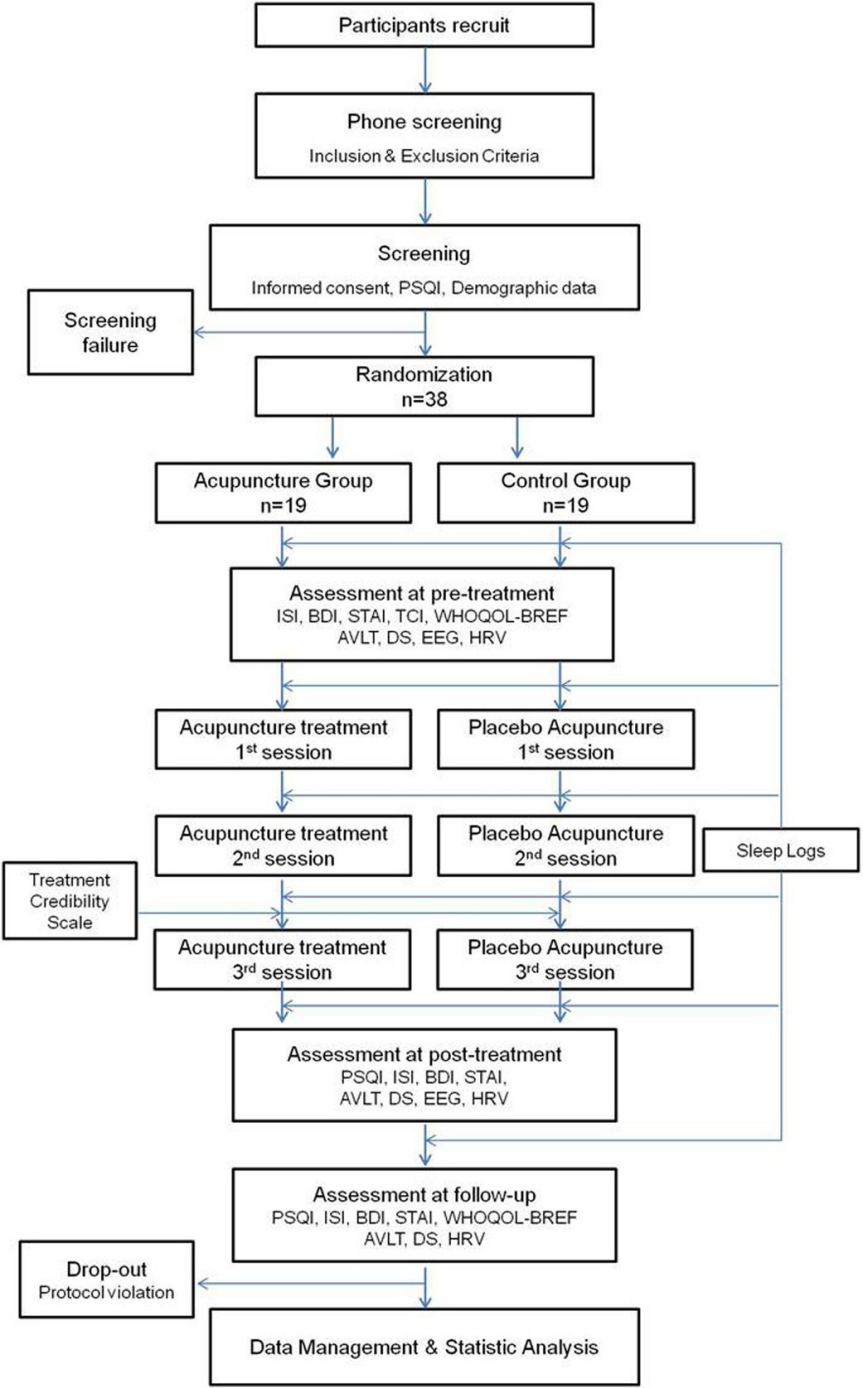
**Study flow chart.**

**Figure 2:**
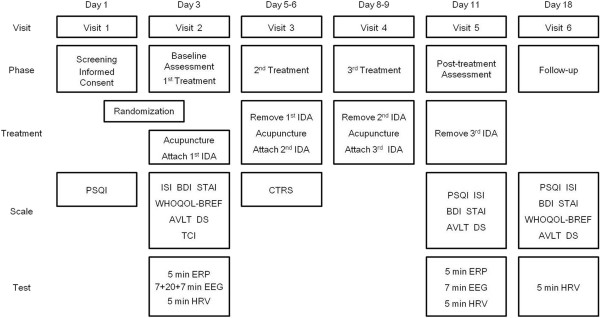
**Treatment and assessment schedule.**

Figure 1 is an overview of this trial, and Figure 2 is an approximate visit schedule.

### Participants

#### Inclusion criteria

- Male or female over 18 under 65 years old
- Reports clinical symptoms for insomnia and score of 6 or above on the PSQI
- Demonstrates no problems with communication, both visual and auditory (reading, writing, hearing, speaking, and watching)
- Signed the written informed consent form for the clinical trial

#### Exclusion criteria

- Has taken Western and/or Oriental medicine and/or dietary supplements in the past 2 weeks for insomnia
- Has a severe neural or psychiatric disorder or a history of major neuropsychiatric disorder (for example, autism, learning disorder, mental retardation, and so on)
- Has received acupuncture for insomnia treatment in the past month
- Has infection or inflammation at the acupoints
- Has a hemorrhagic disease or anticoagulant intake
- Has a serious physical disorder
- Has participated in any other clinical trial within a month of the screening date
- Pregnancy, breast-feeding, or being a woman of childbearing age not on a proper method of birth control

### Assessments

All assessments in the trial are shown in Figure 2.

### Interventions

In the experimental group, patients will receive needle acupuncture treatment on 5 specified acupoints (PC6, SP6, HT7, KI6, and BL62) for 20 minutes. After 20 minutes, the needles will be removed and intradermal acupuncture (IDA) placed at the same points. The smaller IDA shall be kept attached on the skin for 48 to 72 hours and all patients will be instructed to stimulate themselves before sleep.

In the control group, all interventions shall be the same as those of the experimental group except the location of the acupoints, substituting them with five sham acupoints. The sham acupoints shall be about 1.0 cm from the real acupoints in the experimental group and are spots that do not belong to standard acupoints on the body and are not suitable for actual acupuncture based on anatomical and other conditions. IDA will be placed at five sham acupoints in the control group as well with the same instructions as that of the experimental group.

All acupuncture treatment complies with the Standards for Reporting Interventions in Clinical Trials of Acupuncture (STRICTA) revised in 2010 (see Table 1) [29].

**Table 1:** **Interventions details by Standards for Reporting Interventions in Clinical Trials of Acupuncture (STRICTA) items**

Item	Details	Intervention
1. Acupuncture rationale	1a) Style of acupuncture	Traditional needle acupuncture and intradermal acupuncture
	1b) Reasoning for treatment provided based on historical context, literature sources, and/or consensus methods, with references where appropriate	Selected traditional acupuncture points on the 12 meridian system based on the systematic review by Yeung and clinical experiences
	1c) Extent to which treatment was varied	No variation
2. Details of needling	2a) Number of needle insertions per subject per session (mean and range where relevant)	Five needle insertions per 1 session
	2b) Names (or location if no standard name) of points used (uni/bilateral)	*Neiguan* (PC6), *Sanyinjiao* (SP6), *Shenmen* (HT7), *Zhaohai* (KI6), *Shenmai* (BL62), all unilateral.
	2c) Depth of insertion, based on a specified unit of measurement or on a particular tissue level	Depth of needle insertion was at least 5 to10 mm and intradermal acupunctured was 1 to 2 mm
	2d) Response sought	*De-qi* sensation felt by practitioner and subject
	2e) Needle stimulation	No stimulation and just retention without movement Intradermal acupuncture fixed with skin tape and subjects educated to self finger-press on them at nighttime
	2f) Needle retention time	Twenty-minute retention for needles, intradermal acupuncture patched 48 to 72 hours until next session
	2 g) Needle type (diameter, length, and manufacturer or material)	Needles: 0.25 × 40 mm, stainless steel (Dong Bang Medical Co. Ltd, Korea)
		Intradermal acupuncture: 0.18 × 1.3 × 1.5 mm, stainless steel (Dong Bang Medical Co. Ltd, Korea)
3. Treatment regimen	3a) Number of treatment sessions	Three sessions
	3b) Frequency and duration of treatment sessions	Three times per week, interval of 1 to 4 days between sessions
4. Other components of treatment	4a) Details of other interventions administered to the acupuncture group	Sleep hygiene education
	4b) Setting and context of treatment, including instructions to practitioners, and information and explanations to patients	Same practitioner treats every subject at every session in the same outpatient clinic
5. Practitioner background	5) Description of participating acupuncturists (qualification or professional affiliation, years in acupuncture practice, other relevant experience)	Licensed traditional Korean medicine doctor at the Kyung Hee East-West Neo Medical Center at Gangdong with more than 10 years of acupuncture treatment experience
6. Control or comparator interventions	6a) Rationale for the control or comparator in the context of the research question, with sources that justify this choice	Sham acupuncture points
	6b) Precise description of the control or comparison group. If sham acupuncture or any other type of acupuncture-like control is used, provide details as for items 1 to 3 above	Same as intervention group except 2b) and 2d)
		2b) No acupuncture points located away from acupuncture points at least 10 mm
		2d) Without *De-qi* sensation

### Outcomes

#### Primary outcome

The primary outcome of this study will be the changes in the ISI between the baseline, post-treatment assessment, and 1-week follow-up.

The PSQI is an effective self-report questionnaire used to assess the quality of sleep and sleep disturbances. The PSQI is comprised of 19 items consisting of the following components: subjective sleep quality, sleep latency, sleep duration, habitual sleep efficiency, sleep disturbances, use of sleeping medication, and daytime dysfunction. The ISI is a five-point Likert-scale self-rate questionnaire designed to assess the nature, severity, and impact of insomnia, and is widely used to measure responses to treatment in research studies and clinical trials.

#### Secondary outcome

Secondary outcome measures refer to scores on the BDI, STAI, WHOQOL-BREF, K-AVLT, and DS, the weekly average of the components in the sleep logs during the 2-week period, and changes in EEG and HRV between the baseline, post-treatment assessment, and 1-week follow-up (only the EEG was excluded at 1-week follow-up).

The BDI and STAI measure emotional states. The BDI is a self-report scale used in clinics and research commonly for measuring severity of depression through a 21-item multiple-choice questionnaire. The STAI in turn is a 40-question self-report inventory based on a 4-point Likert scale that measures each state and trait of anxiety. Normalized Korean versions of both instruments will be used in the trial. The WHOQOL-BREF will be used for measuring quality of life related to psychological health, social relationships, and environment. It consists of 26 items, a shorter version than the original instrument for research studies and clinical trials. A Korean version of this questionnaire will be used.

The sleep log is a diary to be kept by the patients themselves of their sleeping and waking times as well as additional sleep-related information. It is a useful tool in diagnosing insomnia and monitoring whether treatment is working. Sleep logs include 16 points of information such as sleep-onset latency, waking after sleep onset, time in bed, total sleep time, number of awakenings, sleep quality, and so on.

The K-AVLT is a Korean version of the Auditory Verbal Learning Test normalized for Koreans and is used to evaluate verbal learning and memory abilities in terms of neuropsychological assessment. The DS in turn measures working memory and attention.

EEGs will be recorded from 8 channels (F3, F4, Fz, T3, Cz, T4, Pz and electrooculogram) with QEEG-8 (LXE3208, LAXTHA Inc. Daejeon, Korea). The right earlobe (A1) will be used as a reference electrode during data recording. The electrooculograms (EOG) generated from eye blinks or movements shall be recorded with an electrode placed approximately 1 cm below the subject’s right outer eye can thus. Electro-physiological signals will be amplified (20,000) and filtered (bandpass filter = 0.25 to 50 Hz). EEG signals shall be digitized to 256 Hz.

HRV will be recorded by QECG-3(LXC3203, LAXTHA Inc. Daejeon, Korea) for 5 minutes while the patient maintains a sitting posture.

### Safety assessment

The experiment will ask subjects to report adverse events (AEs), such as unexpected physical changes or side effects, in person when they visit and/or by telephone at other times during the study.

Every adverse event reported by subjects will be described in the case report form (CRF). If the adverse event is severe and associated with the trial, the participant will be withdrawn from the study and given appropriate medical care.

### Sample size

Sample size was calculated based on a ratio of 1:1 between the experimental and control groups. This sample size is based on a study that investigated the effect of acupuncture treatment on insomnia using ISI as a primary outcome [30]. In that study, the mean change in ISI between the experimental and control groups was 3.8 and the standard deviation was estimated as the median between the experimental (SD =4.0) and control groups (SD =3.2). Based on a two-tailed alpha error of 5% and with a statistical power of 80%, the calculated sample size is based on the equation:


Therefore, all 38 participants will be randomly assigned with 19 subjects in each group, assuming a 20% dropout rate.

### Randomization

An expert will randomly generate a block size (4 or 6) sequence using Microsoft Excel (Redmond, WA, USA). After that, with the allocation ratio of the experimental and control group within each block kept at 1:1, the expert will randomly generate the numbers 0 (control group) or 1 (experimental group) through Microsoft Excel. This expert will not contact any of the patients during the experiment. Once the expert generates the random numbers, the expert will seal each number in an opaque envelope. The expert lets the clinician know each random number as the clinician calls a patient by telephone. Two other experts (neuropsychologists) - who measure the self-reported tests, the psychological (cognitive) tests, the EEG and HRV - will be blinded. Only the clinician who treats acupuncture therapy knows what treatment the patient has been administered, but he is prohibited from accessing any measurements for outcomes. Double-blinding is almost impossible due to the specifications of the acupuncture treatment. Thus, in this trial, only the neuropsychologist (assessor), the statistical expert, and patients will be blinded.

### Statistical method

The primary outcome variable for efficacy analysis is ISI, and secondary outcomes are the PSQI, BDI, STAI, WHOQOL-BREF, sleep logs, K-AVLT, DS, EEG, and HRV. Demographics, sleep logs, ISI, PSQI, K-AVLT, Digit Span, STAI, TCI, EEG, and HRV will be analyzed using independent sample *t*-tests at baseline to determine equality of the two groups. A chi-square test will be used to assess sex differences. An efficacy analysis will be done using both per-protocol and intention-to-treat analyses.

Independent *t*-tests between the two groups and a repeated-measures analysis of variance (ANOVA) will be used to determine and compare the effect of acupuncture. The factors are treatment (3 levels: pre-, post-acupuncture and 1-week follow-up) and groups (2 levels: treatment and sham) and their interaction. The significance level was set at *P <*0.05 and *post-hoc* analyses were performed where appropriate.

### Monitoring

A qualified clinical trial expert will monitor this study. This trial in particular will be monitored by Gajin Han, OMD, PhD, a research professor at the College of Korean Medicine, Kyung Hee University. Han is a professional clinical research associate, having completed a formal training program organized by the Korea National Enterprise for Clinical Trials. Monitoring will commence after the first participant completes the entire period of this study.

### Ethical considerations

The Institutional Review Board (IRB), prior to subject recruitment, has already approved the entire design and procedure of the clinical trial, CRF, and all measures that will be used in the study (reference number KHNMC-OH-IRB 2011-015, IRB at Kyung Hee University Gangdong Medical Center, approved on the 13 September 2012). All subjects must provide written informed consent prior to study participation.

The trial will be performed in accordance with the principles of good clinical practice of the Korean Food and Drugs Administration and/or the Declaration of Helsinki 2008.

## Discussion

Standards for Reporting Interventions in Clinical Trials of Acupuncture (STRICTA) were revised in 2010, yet few studies have rigorously observed these recommendations. Consequently, the results of many previous studies likely do not meet standards of objectivity and methodology. In our proposed study, all details such as acupoints, verification tools, and use of acupuncture needles and procedures will be reported objectively following STRICTA standards.

Previous trials of acupuncture treatment have focused only on subjectively reported symptoms of insomnia. Consequently, they are not able to observe the mechanisms of acupuncture’s effect on insomnia. In the present trial, HRV will be used to assess changes in the autonomic nervous system and EEG will be used to assess changes in brain activity in real time. These measures will objectively examine physiological changes due to acupuncture in real time.

Depression, anxiety, and decline in cognitive functions are the main consequences of chronic insomnia as reported by many previous studies, and such symptoms ultimately degrade quality of life. The effect of acupuncture on insomnia will be verified at multi-dimensional levels in this trial, assessing psychological and emotional states, cognitive functions, and quality of life, allowing us to observe how the relief of insomnia symptoms due to acupuncture treatment improves patients’ quality of life.

In the trial, we will test the intradermal acupuncture method particularly for enhancing the effectiveness of acupuncture for insomnia. The acupuncture is stimulated every night by subjects applying it continuously on their own, even at home.

The design of the control group in studies verifying the effect of acupuncture treatment is always an issue. For this blind-controlled study, STRICTA suggests active acupuncture control, penetrating needle control, or non-penetrating sham needling control. The present proposed trial will use 'sham’ acupuncture points as active acupuncture control. However, it is possible that sham acupuncture may lead to improvement. Such a methodological limitation should be improved upon in future studies. A double-blind trial is not possible because of the particular treatment method of the controls. This limitation will be addressed with an evaluation by an experimenter who is not involved in the treatment. Furthermore, there are limitations associated with the lack of polysomnographic data due to constraints of time, expense, and space in the hospital, as the present trial is still a pilot study. The actigraphy and polysomnography are objective tools for assessing insomnia and are also highly recommended, but many other studies have used them as secondary outcomes while still others did not use actigraphy and/or polysomnography as outcomes at all [31–33, 10, 11]. However, polysomnography will be assessed in our planned future expanded trials.

## Trial status

This clinical trial is currently recruiting participants.

## Abbreviations

AEs: adverse events
ANCOVA: analysis of covariance
BDI: Beck Depression Inventory
CRF: case report form
CTRS: Credibility of Treatment Rating Scale
DS: Digit Span Test
DSM-IV: The Diagnostic and Statistical Manual of Mental Disorders, Fifth Edition
EEG: electroencephalogram
ERPs: Event Related Potentials
HF: high frequency
HRV: heart rate variability
IDA: intradermal acupuncture
IRB: Institutional Review Board
ISDC-2: International Classification of Sleep Disorders, Second Edition
ISI: Insomnia Severity Index
K-AVLT: Korean-Auditory Verbal Learning Test
LF: low frequency
PSQI: Pittsburgh Sleep Quality Index
STAI: State-Trait Anxiety Inventory
STRICTA: Standards for Reporting Interventions in Clinical Trials of Acupuncture
TCI: Temperament Character Inventory
WHOQOL-BREF: World Health Organization Quality of Life Abbreviated Version.
